# Direct warming and rehydration method in frozen embryo transfer: study protocol for a pragmatic, multi-center, double-blinded, two-arm randomized controlled trial

**DOI:** 10.1186/s13063-026-09663-x

**Published:** 2026-05-18

**Authors:** Waner Wu, Murong Xu, Ka Kei Fung, Linyao Zhang, Hoi Ming Wan, Carol Pui Shan Chan, Jacqueline Pui Wah Chung, David Yiu Leung Chan

**Affiliations:** 1 Assisted Reproductive Technology Unit, Department of Obstetrics and Gynaecology, The Chinese University of Hong Kong, Hong Kong SAR, China; 2 School of Biomedical Science, The Chinese University of Hong Kong, New Territories, Hong Kong SAR, China

**Keywords:** Cryopreservation, Embryo warming, Vitrification, Direct warming, Live birth rate, Cost-effectiveness

## Abstract

**Background:**

Embryo cryopreservation through vitrification is widely used to store surplus embryos for future transfer. Traditional multi-step warming methods are both time-consuming and costly. This study evaluates the clinical outcomes and the effectiveness of a novel direct warming method for frozen embryo transfer (FET) in comparison to the conventional multi-step method.

**Methods:**

This is a pragmatic, two-arm, double-blinded randomized controlled trial (RCT) comparing the new direct warming method with the conventional multi-step method. Participants undergoing FET will be randomly assigned to either the direct warming method or the conventional warming protocol. The primary outcomes are the clinical pregnancy rate (CPR), ongoing pregnancy rate (OPR), the live birth rate (LBR), miscarriage rate, and cost-effectiveness. Secondary outcomes include identifying clinical differences among various brands of warming media, embryo storage devices, and embryo culture media through subgroup analyses. A sample size of 578 participants is planned, allowing for a 5% loss to follow-up.

**Discussion:**

Preliminary studies have shown that the direct warming method offers comparable or slightly higher live birth rates than conventional methods. Moreover, it significantly reduces thawing time and associated costs, offering a faster, cost-effective option for FET. This trial has the potential to improve accessibility to ART by reducing procedural costs and increasing efficiency without compromising clinical outcomes.

**Trial registration:**

ClinicalTrials.gov NCT06741748. Registered on 19 November 2024.

## Introduction

### Background and rationale {6a}

Infertility affects approximately one in six individuals globally, making assisted reproductive technologies (ARTs) essential for many couples attempting to achieve pregnancy [1]. Among ART procedures, frozen embryo transfer (FET) has become the most frequently performed technique, surpassing 2 million cycles annually since the first live birth achieved via frozen embryo transfer using slow freezing [2, 3]. Vitrification has since emerged as the dominant method for embryo cryopreservation, significantly improving survival rates compared to the earlier slow-freezing method [4]. Although vitrification has replaced slow freezing, the corresponding advancements in embryo warming techniques have not progressed as rapidly.

The process of thawing vitrified embryos requires precise and rapid warming rates, specific thawing solutions, and the expertise of skilled embryologists to ensure optimal survival rates [5]. Conventionally, the multi-step warming process gradually dilutes and removes cytotoxic cryoprotectants, maintaining extracellular osmolality to avoid osmotic shock. However, this method typically requires 15–20 min and incurs significant costs, approximately 5500 HKD per procedure. Not only is this multi-step approach costly, but it also extends the time required to prepare embryos for transfer.

Our research introduces a novel direct warming method that offers a faster, cost-effective alternative. Unlike other ultra-fast warming methods, which utilize sucrose or trehalose in thawing solutions, our approach employs embryo culture mediums without any cryoprotectants [6–8]. This method has demonstrated a 100% survival rate in over 100 donated human blastocysts. The entire warming process is completed in just 1 min, reducing the time required by 90% and lowering costs by 80% [9].

This innovative method challenges conventional perceptions of embryo warming. While vitrification has revolutionized cryopreservation, leading to improved safety and clinical outcomes for frozen embryo transfers, the embryo warming process has lagged in comparison [10]. Current laboratory protocols rely on commercial thawing kits that involve multiple steps and specific cryoprotectants [11]. Among these, ultra-fast warming methods using cryoprotectants like sucrose or trehalose are commonly employed to manage osmotic shock during embryo warming [5–7]. While these methods are effective, they involve multiple steps and carry the risk of residual toxicity from the cryoprotectants. In contrast, our direct warming method eliminates the need for cryoprotectants, providing a simpler approach that controls the risk of osmotic stress, avoids cryoprotectant-related toxicity, and reduces potential handling errors. This method not only speeds up the warming process and lowers material costs but also improves consistency and reproducibility, making it a more efficient and reliable alternative in clinical practice.

The aim of this study is to show the direct warming method is at least equivalent to the conventional method plus can save billions of dollars from patients with the same healthy take-home baby rate.

### Objectives {7}

#### Primary objective

The primary objective is to evaluate the clinical outcomes and cost-effectiveness of the novel direct warming method compared to the conventional multi-step warming method for FET in terms of clinical pregnancy rate (CPR), ongoing pregnancy rate (OPR), live birth rate (LBR), miscarriage rate, and time efficiency, cost-effectiveness.

#### Secondary objective

The secondary objective is to evaluate the impact of technical variations (warming media, storage devices, and culture media) on clinical outcomes between the direct warming method and the conventional multi-step method.

### Trial design {8}

This is a two-arm, double-blinded, parallel-group, multi-center, randomized controlled trial (RCT) with a superiority framework. The trial will compare the efficacy and safety of the new direct warming method against the conventional multi-step warming method for FET.

Participants will be randomized in a 1:1 allocation ratio into one of two groups:

1. Intervention (arm 1): the new direct warming method.
2. Control (arm 2): standard care using the conventional multi-step warming method.

## Methods: participants, interventions, and outcomes

### Study setting {9}

This study will be conducted at:

1. IVFHK-CUHK, the leading in vitro fertilization (IVF) center of this trial. The center situates in the Prince of Wales Hospital (PWH) and operates under the Department of Obstetrics and Gynaecology at The Chinese University of Hong Kong (CUHK).
2. CUHK Medical Centre (CUHKMC), a private IVF center in Hong Kong under the Faculty of Medicine, The Chinese University of Hong Kong.
3. HEAL Fertility, a private IVF center in Hong Kong.

Other IVF centers may be allowed to join this trial.

Participants for the study will be recruited from patients undergoing FET cycles at these sites. Detailed information will be provided verbally and through consent forms, with eligibility criteria checked before inclusion in the study. Recruitment will be managed by the IVF research team and clinical team at PWH. Data collection will occur only at this site, and no international sites are involved in this local trial.

### Eligibility criteria {10}

#### Inclusion criteria

Participants will be eligible for inclusion in this study if they meet the following criteria:

1. Patients undergoing a FET cycle at one of the study centers.
2. Plan to undergo single embryo transfer (SET) during the FET cycle.
3. Age between 18 and 45 years.
4. Patients with at least one good-quality blastocyst suitable for transfer.
5. Patients who have provided informed consent to participate in the study.

#### Exclusion criteria

Patients will be excluded from the study if they meet any of the following criteria:

1. Patients with repeated implantation failure (RIF) or recurrent miscarriage (RM).
2. Patients with known uterine anomalies or significant uterine pathology (e.g., fibroids, polyps).
3. Patients who are unwilling or unable to provide informed consent.

### Who will take informed consent? {26a}

The informed consent for this trial will be conducted by a designated research assistant, nurse, or clinician at any of the participating study sites. The research assistant will be responsible for providing potential participants with comprehensive information regarding the study. This will include an explanation of the study’s purpose, procedures, potential risks, and benefits, as well as the rights of participants.

### Additional consent provisions for collection and use of participant data and biological specimens {26b}

There is no collection of biological specimens planned, and thus no additional consent will be required for biological samples.

## Interventions

### Explanation for the choice of comparators {6b}

In this trial, the comparator chosen is the conventional multi-step warming method, which is the current standard of care for thawing vitrified embryos in ART procedures [12]. This method has been widely used in clinical practice due to its established safety and effectiveness, making it an appropriate comparator for evaluating the new direct warming method.

The conventional multi-step method involves a series of steps that gradually remove cryoprotectants and rehydrate the embryo, ensuring its post-thaw survival. It typically requires 15–20 min to complete and is performed by trained embryologists. Despite its widespread use, the multi-step process is time-consuming and can incur significant costs, making it a useful benchmark for comparison with the new direct warming method, which aims to simplify the procedure and reduce both time and cost while maintaining or improving clinical outcomes.

The choice of this comparator allows the study to directly assess whether the new direct warming method can offer a more efficient and cost-effective alternative without compromising the success rates of embryo survival, implantation, pregnancy, and live birth.

### Intervention description {11a}

1. Intervention group (arm 1): new direct warming method
  In the intervention group, vitrified blastocysts will be thawed using a novel direct warming method. The ready-to-be-thawed blastocyst is placed in a pre-warmed direct warming medium for 1 min. Following this, the embryo is transferred directly into the embryo culture medium in a time-lapse system until it is ready for transfer into the uterus. This new warming method significantly reduces the procedure time to approximately 3 min, making it much faster than the conventional multi-step warming process.
2. Control group (arm 2): conventional multi-step warming method
  The control group will follow the conventional multi-step warming method. In this procedure, vitrified embryos are first placed in a pre-warmed thawing solution for 1–3 min. They are then transferred to a dilution solution for 4–6 min to gradually dilute the cryoprotectants, followed by immersion in a washing solution for 5–10 min to complete the rehydration process. This multi-step method takes approximately 20 min to complete. After thawing, the embryos are placed in the embryo culture medium in a time-lapse system until they are ready for transfer.
  Blastocysts will be evaluated using the Gardner grading scale with a three-part criterion on blastocyst expansion, inner cell mass (ICM), and trophectoderm (TE) quality. Blastocysts with a score ≥3BB are considered good quality [13].

### Criteria for discontinuing or modifying allocated interventions {11b}

1. Participant request:
  If a participant requests to withdraw from the study at any time, their allocated intervention will be discontinued. The participant will be informed that they may withdraw without any negative consequences, and their withdrawal will not affect their future clinical care.
2. Medical conditions:
  Should a participant’s medical condition change during the trial (e.g., significant changes in uterine health, onset of illness that may affect pregnancy outcomes), the intervention may be modified or discontinued in consultation with the participant and the study’s clinical team.
3. Safety concerns:
  If any safety concerns arise during the embryo warming or transfer process, such as adverse reactions or unforeseen complications affecting the embryo’s viability or the participant’s health, the intervention may be halted. The trial’s clinical staff will monitor participants closely and stop the procedure if it is deemed unsafe to continue.

### Strategies to improve adherence to interventions {11c}

1. Training and standardization:
  All embryologists and clinical staff involved in the intervention and control procedures will receive detailed training on the standardized protocols for both the conventional multi-step warming method and the new direct warming method. This will ensure that all procedures are carried out consistently across participants and in line with the trial protocol.
2. Regular monitoring:
  A dedicated Trial Monitoring Team will be established to observe and ensure protocol adherence throughout the trial. They will periodically review the warming and embryo transfer procedures, both in the intervention and control groups, to identify any deviations from the protocol.
3. Documentation of procedures:
  For every participant, a detailed record of the warming process will be maintained, documenting the exact steps, time taken, and any deviations from the protocol. This will allow for tracking of adherence and enable prompt corrective actions if any protocol deviations are noted.
4. Adherence audits:
  Routine audits will be conducted at regular intervals to review all aspects of the trial, including adherence to intervention protocols. These audits will ensure that any potential issues in protocol adherence are identified early and addressed.
5. Participant engagement:
  Participants will be closely monitored for any concerns or discomfort during the procedure. Regular follow-ups will be scheduled. Any issues will be addressed by the clinical team immediately.
  By implementing these strategies, the trial aims to ensure high adherence to both the intervention and control protocols while minimizing variability in the procedures administered across the study.

### Relevant concomitant care permitted or prohibited during the trial {11d}

#### Permitted concomitant care

1. Standard fertility treatments:
  Participants may continue with standard fertility treatments such as hormonal support (e.g., progesterone, estrogen) as part of their IVF protocols. These treatments are necessary to maintain the optimal environment for embryo implantation and pregnancy support. The handling clinician will determine suitable treatment for the patients.
2. Routine medications:
  Participants can continue any routine medications for pre-existing conditions (e.g., medications for asthma or mild allergies), provided these do not interfere with the study outcomes. The use of such medications will be documented for each participant.
3. Follow-up appointments:
  Regular follow-up visits, including ultrasound scans and blood tests, are part of routine IVF care and will be permitted as part of the standard treatment plan.

#### Prohibited interventions

1. Experimental fertility treatments:
  Any unapproved or experimental fertility treatments or medications that are not part of the standard IVF protocol will be prohibited during the trial.
2. Unapproved medications or supplements:
  Participants will not be allowed to take any fertility-related medications or supplements (e.g., herbal remedies, unapproved hormonal therapies) that are not prescribed by their reproductive specialist as part of the trial.
3. Other assisted reproductive procedures:
  Participants will be prohibited from undergoing any additional assisted reproductive procedures (e.g., intrauterine insemination, gamete donation) outside of the planned embryo transfer during the trial.
  All concomitant care and interventions will be closely monitored, and any deviations from the allowed procedures will be documented. The trial’s clinical team will review these cases to assess any potential impact on the study outcomes.

### Provisions for post-trial care {30}

#### Ancillary and post-trial care

Participants will continue to receive routine clinical care as part of their standard fertility treatment at the PWH following their participation in this trial. This includes all necessary follow-up visits to monitor pregnancy outcomes and any standard care associated with IVF or embryo transfer. If any minor adverse events or complications related to the embryo warming process are encountered, appropriate medical care will be provided by the clinical team at no additional cost to the participant.

It is important to note that the most severe outcome related to embryo warming in this trial is likely to be the early cessation of embryo development, which may affect the overall pregnancy success rate. Studies have shown that miscarriage rates after FET are generally comparable to those with fresh transfers, provided the thawed embryo is healthy and viable [14, 15]. Furthermore, other ultra-fast warming methods have not shown an increased risk of miscarriage or adverse effects on pregnancy outcomes [7, 8]. No major clinical events, such as miscarriage or significant health complications, are expected to result directly from the embryo warming process, as warming itself does not significantly increase the risk of miscarriage.

#### Compensation for harm

Given the low-risk nature of the embryo warming procedure, particularly in relation to the embryo’s viability and pregnancy success rates, the likelihood of serious harm to participants is extremely low. However, in the unlikely event that a participant experiences an adverse event directly caused by the procedures involved in the trial, the study has comprehensive insurance coverage in place to provide compensation.

Participants will be informed during the informed consent process that they will not receive monetary compensation for participation in the trial itself. However, any medical costs related to adverse events caused by trial-related procedures will be fully covered by the study’s insurance policy. This ensures that participants are protected and that any necessary medical care will be provided at no cost to them.

### Outcomes {12}

#### Primary outcomes

The primary outcome of this study is to evaluate the clinical efficacy of the new direct warming method for FET compared to the conventional multi-step warming method. The key measures for the primary outcome include:

1. Clinical pregnancy rate: defined as the presence of at least one intrauterine gestational sac detected via ultrasound at 6–8 weeks of gestation. Multiple pregnancies (two or more gestational sacs) are counted as one clinical pregnancy.
2. Ongoing pregnancy rate: defined as the proportion of pregnancies that progress beyond 6–8 weeks, with at least one viable fetus with a heartbeat confirmed via ultrasound.
3. Live birth rate: the proportion of participants who achieve a live birth, defined as the delivery of a live baby after 24 weeks of gestation.
4. Miscarriage rate: the proportion of pregnancies resulting in pregnancy loss after the confirmation of clinical pregnancy.
5. Cost-effectiveness: an evaluation of the cost savings achieved by using the new method, including reductions in time, materials, and labor compared to the conventional method.
  - The equation for consumable cost: (total consumable cost of conventional warming in all centers/total cycle number of the conventional warming in all centers)/(total consumable cost of direct warming in all centers/total cycle number of the direct warming in all centers) × 100%.
  - The equation for time cost: (total minutes spent on conventional warming in all centers/total cycle number of the conventional warming in all centers)/(total minutes spent on direct warming in all centers/total cycle number of the direct warming in all centers) × 100%.
  - The equation for labor cost: (total hours spent on conventional warming in all centers/total cycle number of the conventional warming in all centers * role-specific hourly rates)/(total hours spent on direct warming in all centers/total cycle number of the direct warming in all centers * role-specific hourly rates) × 100%.

#### Secondary outcomes

The secondary outcomes include additional clinical and operational metrics, assessing both the effectiveness of the warming method and its broader impact on the ART process:

1. Warming media comparison: evaluating embryo survival, blastocyst re-expansion, and implantation success across different brands.
2. Embryo storage devices: assessing the impact of various storage devices on post-thaw embryo viability and clinical pregnancy rates.
3. Embryo culture media: comparing embryo development, implantation potential, and clinical outcomes using different culture media.

All outcomes will be measured at specific time points relevant to the embryo warming, transfer, and post-transfer processes, including follow-ups to 6–8 weeks for clinical pregnancy and further monitoring for live birth.

### Participant timeline {13}

**Fig. 1:**
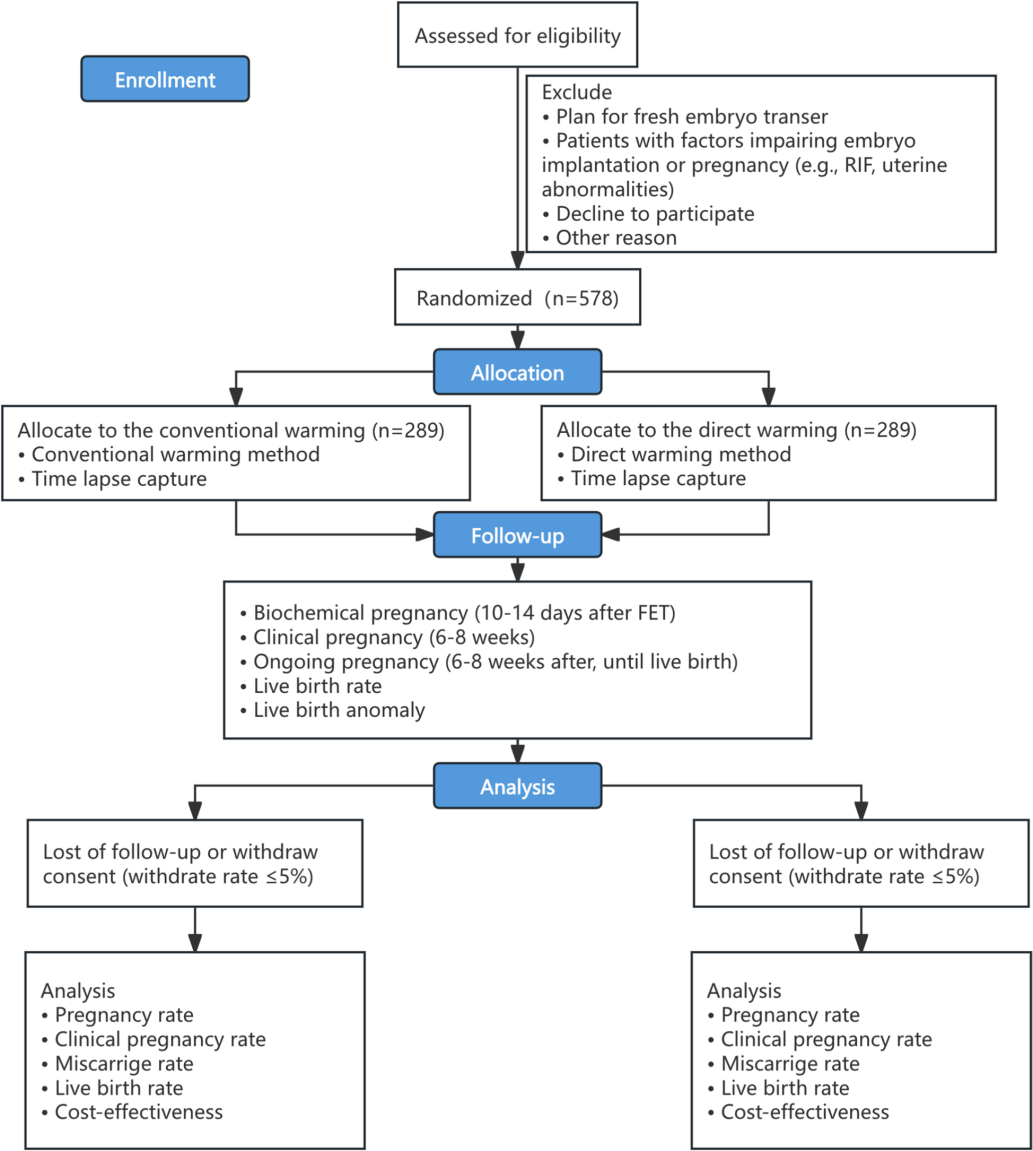
RCT flowchart

See Fig. 1 and Table 1.

**Table 1:** Schedule of the study process

Timepoint	Study period							
	Enrollment	Allocation	Post-allocation					Close-out
	IVF talk	0 week	Embryo transfer	10–14 days	6–8 weeks	20–24 weeks	36–40 weeks	6–8 weeks after birth
Enrollment								
Eligibility screen	X							
Informed consent	X							
Allocation		X						
Interventions								
Administration of different embryo warming methods			X					
Assessments								
Post-thaw embryo assessment			X					
HCG test				X				
Ultrasound of gestation					X			
Phone follow-up						X	X	X

### Sample size {14}

The sample size calculation was based on preliminary data indicating approximately a 5% increase in the live birth rate with the new method compared to conventional warming. With type I error (*α*) set at 0.05 and type II error (*β*) set at 0.2 (80% power), and assuming a clinically significant difference of 20% in the worldwide clinical pregnancy rate, the required sample size per arm is 275, calculated using MedCalc 15.6.1. To account for a 5% loss to follow-up, a total of 578 participants will be enrolled (289 per arm). Given that the total number of participating centers is currently undetermined, the leading center, IVFHK-CUHK, will serve as the core recruitment site. This center can independently recruit at least 578 participants.

### Recruitment {15}

This trial will recruit participants from the joint study sites listed above. IVFHK-CUHK has extensive experience in conducting large-scale clinical trials, including a published international multi-center RCT on embryo time-lapse imaging [16]. The study has enhanced recruitment strategies and operational efficiency at both centers. With a strong infrastructure and prior success in participant enrollment, this trial is well-positioned to achieve the target sample size efficiently.

## Assignment of interventions: allocation

### Sequence generation {16a}

A stratified randomization approach will be used to ensure balanced distribution of participants across treatment groups based on age, a key factor influencing embryo warming and transfer outcomes. Participants will be stratified into two age groups: ≤35 years and >35 years.

Within each age stratum, participants will be randomly assigned to one of two study groups (conventional warming or direct warming) using a computerized randomization process in sealedenvelope.com. Random numbers will be generated for each participant, and participants will be assigned to the intervention or control group randomly, ensuring even distribution across groups within each age stratum.

In the case of an odd number of participants in any stratum, the remaining participant will be randomly assigned to one of the two groups. This method ensures that participants are randomized within each stratum, achieving a balance in age distribution across the treatment groups.

### Concealment mechanism {16b}

The allocation sequence will be concealed using a sequentially numbered, opaque, sealed envelope system. After stratified randomization has been conducted in sealedenvelope.com, each participant’s group assignment will be placed in a numbered, opaque envelope. These envelopes will be prepared in advance and will only be opened by the embryologist responsible for embryo handling. The research staff/nurse/clinician responsible for enrolling participants will not be aware of group assignments, ensuring allocation concealment until the interventions are carried out.

After the embryo transfer, if it is determined that the patient is not pregnant, their group assignment may be disclosed to them. However, if pregnancy is confirmed, the group assignment will remain concealed, and the patient will not be informed until a live birth occurs, or in the event of a miscarriage or elective termination. This approach ensures allocation concealment throughout the pregnancy, minimizing bias and maintaining the integrity of the trial.

### Implementation {16c}

The research staff will be responsible for enrolling participants. After participants are enrolled, each participant’s group assignment will be determined by opening a sequentially numbered, opaque, sealed envelope, which will have been prepared in advance by the research staff following the randomization process. The envelopes will only be opened after baseline data collection is complete to ensure allocation concealment.

## Assignment of interventions: blinding

### Who will be blinded {17a}

This trial will be conducted as a double-blinded study. Both the trial participants and the clinical team will be blinded to the group assignments after the allocation to interventions has been completed. Neither group will have access to information about whether participants are in the intervention group (direct warming method) or the control group (conventional warming method).

Outcome assessors will also be blinded to the group assignments to ensure unbiased evaluation of primary and secondary outcomes, including embryo survival rates, pregnancy rates, and live birth rates. Data analysts will be blinded to the group allocation during the analysis phase to prevent bias in data interpretation.

The use of sequentially numbered, opaque, sealed envelopes ensures allocation concealment until the point of intervention, maintaining blinding throughout the trial.

Embryologists cannot be blinded in this trial.

### Procedure for unblinding if needed {17b}

This trial is designed to be double-blinded; however, unblinding will be permitted only in cases where it is necessary for participant safety or when a serious adverse event occurs that requires knowledge of the participant’s group allocation to guide clinical care.

If unblinding is required, the following procedure will be followed:

1. The clinical team responsible for the participant’s clinical management will contact the principal investigator (PI).
2. The PI will review the case and determine if unblinding is necessary.
3. If unblinding is deemed essential, the allocation code will be revealed to the care provider.
4. The unblinding event, reason for unblinding, and any actions taken will be documented in the trial records.

Routine unblinding or revealing of group allocations will not occur unless absolutely necessary for participant safety.

## Data collection and management

### Plans for assessment and collection of outcomes {18a}

The assessment and collection of outcomes, baseline, and other trial data will follow standardized procedures to ensure accuracy and consistency. The primary and secondary outcomes, including pregnancy rates, live birth rates, and embryo survival rates, will be assessed through clinical evaluations, ultrasound examinations, and laboratory testing.

Baseline data will be collected at the time of enrollment, including participants’ age, fertility history, and any relevant medical conditions. These data will be collected using structured case report forms (CRFs), designed to minimize errors and ensure consistency across participants. The CRFs will be attached as part of the study protocol for reference.

Study instruments, such as ultrasound machines for clinical pregnancy assessment and standardized laboratory assays for hCG measurements, will be used. These instruments have been validated for reliability and accuracy in clinical settings.

All data will be recorded in a secure, encrypted electronic database. Regular data audits will be performed to ensure completeness and accuracy.

### Plans to promote participant retention and complete follow-up {18b}

To promote participant retention and ensure complete follow-up, the research team will maintain regular contact with participants through phone calls. Participants will be scheduled for follow-up assessments at specific time points post-embryo transfer, including hCG testing 2 weeks after transfer, ultrasound at 6–8 weeks gestation, and follow-up on pregnancy outcomes such as live birth and birth abnormalities.

For participants who discontinue or deviate from the intervention protocols, efforts will be made to collect all relevant outcome data up until the point of withdrawal. This will include:

1. Reason for discontinuation: collected through phone interviews or in-person discussions.
2. Pregnancy outcomes (if applicable): including any positive or negative pregnancy tests, clinical pregnancy, and live birth outcomes, if the participant is still willing to provide this information.

In cases where participants cannot complete the entire follow-up protocol, efforts will be made to collect as much data as possible from medical records.

### Data management {19}

The study will ensure data integrity and confidentiality through secure and standardized procedures across data entry, coding, storage, and security measures.

1. Data entry: structured CRFs will guide research staff in entering data into a secure, encrypted electronic database. Built-in validation checks will minimize errors, and for critical variables like pregnancy outcomes, double data entry will be implemented to enhance accuracy.
2. Data coding: participants will be anonymized using unique identification numbers. The dataset used for analysis will only contain coded data, with personal identifiers fully removed to protect confidentiality.
3. Data security: strict access controls will be implemented, ensuring that only authorized research personnel can access the data. The encrypted data will be stored on password-protected institutional servers that comply with security standards. Regular data backups will prevent potential data loss.
4. Data storage: data, both physical and electronic, will be securely stored for a minimum of 10 years post-study. Physical consent forms will be kept in locked, secure offices, and electronic data will be encrypted and securely maintained. Personal data will be destroyed according to institutional protocols after the retention period.

### Confidentiality {27}

Personal data of participants will be protected with strict confidentiality throughout the study.

1. Collection: identifiable data, such as contact details and medical history, will be collected during enrollment and kept separate from the anonymized study data. Each participant will be given a unique identification number to ensure anonymity.
2. Sharing: only authorized personnel will have access to identifiable information, and no such data will be shared externally. Any data shared for analysis or reporting will be anonymized to maintain participant confidentiality.
3. Storage: identifiable data will be stored in password-protected, encrypted databases, while physical documents will be securely stored in locked cabinets. Only designated staff will have access to these records.
4. Post-trial: personal data will be stored for a period of 10 years, in line with regulatory guidelines. Once this period has passed, the data will be securely destroyed according to institutional disposal policies.

### Plans for collection, laboratory evaluation, and storage of biological specimens for genetic or molecular analysis in this trial/future use {33}

In this trial, no biological specimens will be collected specifically for genetic or molecular analysis.

## Statistical methods

### Statistical methods for primary and secondary outcomes {20a}

All analyses will follow an intention-to-treat (ITT) approach, with statistical significance set at *p* < 0.05 and 95% confidence intervals (CI). Primary outcomes, including embryo survival rate, pregnancy rate, clinical pregnancy rate, and live birth rate, will be analyzed using logistic regression models, adjusting for potential confounders such as cause of infertility, previous ART cycle, and embryo quality. Chi-square tests or Fisher’s exact tests will be used for categorical outcomes like embryo survival, and logistic regression will report odds ratios (OR) for pregnancy and live birth rates.

Secondary outcomes, including implantation rate and multiple gestation pregnancy rate, will be analyzed using generalized linear models (GLM) and chi-square tests. Thawing time will be compared using *t*-tests or Mann-Whitney *U* tests for non-normal data. Cost-effectiveness will be evaluated through cost-effectiveness analysis (CEA), while post-thaw embryo quality will be assessed using standard grading criteria and analyzed with chi-square tests. Missing data will be handled using multiple imputation to maintain the integrity of the results. An extra sensitivity analysis will be conducted if loss to follow-up is more than 5%.

### Interim analyses {21b}

Given the multi-center nature of this study and the established safety profile of the intervention, no formal Endpoint Adjudication Committee (EAC) or Data Monitoring Committee (DMC) will be established. Instead, the oversight responsibilities for interim analyses and stopping guidelines will be managed by the Trial Steering Committee (TSC).

An interim analysis will be conducted when 50% of the anticipated sample size has been completed, or midway through the trial if any significant safety concerns or deviations from expected outcomes arise. The TSC will review interim results and decide whether to continue, modify, or terminate the trial based on predefined stopping criteria, including serious adverse events directly resulting from the intervention and clear evidence of futility or overwhelming efficacy, such as a 10% embryo survival rate lower than the conventional group. The study team will remain blinded, with only TSC members having access to interim data to ensure unbiased trial conduct.

### Methods for additional analyses (e.g., subgroup analyses) {20b}

Subgroup analyses will be conducted to evaluate the effects of the intervention within predefined groups, such as age (categorized into ≤35 years and >35 years), embryo quality, and cause of infertility. These analyses will help determine if the efficacy of the direct warming method varies across these specific subgroups compared to the conventional multi-step method.

Adjusted analyses will be performed using logistic regression to control for potential confounders, including maternal age, previous IVF cycles, and embryo grading. These adjusted analyses will provide more accurate estimates of the treatment effect on the primary and secondary outcomes.

No additional exploratory analyses are currently planned, though further analyses may be considered based on emerging data trends during the study.

### Methods in analysis to handle protocol non-adherence and any statistical methods to handle missing data {20c}

The primary analysis will be conducted on an ITT basis, which includes all participants as randomized, regardless of protocol adherence or deviations. Participants who discontinue the intervention will still be analyzed in the group to which they were originally assigned to preserve the randomization process.

To address potential missing data, multiple imputation techniques will be employed. Missing values will be imputed based on available baseline and follow-up data, ensuring that the analysis maintains sufficient statistical power. Sensitivity analyses will also be performed to assess the robustness of the results, comparing complete case analyses to those using imputed data.

Per-protocol analyses may also be conducted as a secondary analysis to assess the outcomes among participants who fully adhered to the study protocol.

### Plans to give access to the full protocol, participant-level data, and statistical code {31c}

The full study protocol, participant-level datasets, and statistical code will be made available upon reasonable request to the principal investigator after the study’s conclusion and publication of the main findings. Access to data will be granted in accordance with applicable institutional and regulatory guidelines, ensuring the protection of participant confidentiality.

Data sharing requests will be evaluated on a case-by-case basis, and appropriate data-sharing agreements will be required. Anonymized participant-level data will be provided to ensure that no personal identifiers are shared.

There are no plans for public data repositories or direct public access to the full dataset or statistical code at this time.

## Oversight and monitoring

### Composition of the coordinating center and trial steering committee {5d}

The coordinating center for this trial is based at IVFHK-CUHK, which will oversee the day-to-day management of the study across all participating sites, including CUHK Medical Centre and the HEAL Fertility. The coordinating center is responsible for protocol implementation, site coordination, data collection, monitoring, and regulatory compliance. It will provide logistical and administrative support to ensure adherence to study procedures and timelines. It consists of a trial manager (oversee the entire trial operation, monitor performance indicators, and communicate with other bodies), regulatory specialist (manage and monitor all regulatory documents including ethics application and renewal, protocol amendments, and other reports), site coordinators (communicate with individual centers, especially about the standard procedures), research nurses (provide clinical oversight and review medical records of the participants), and data coordinators (data collection, entry, and storage). The coordinating center will hold regular meetings (at least quarterly) to review trial progress and address operational challenges.

The TSC will provide oversight, ensuring the trial adheres to the study protocol and relevant regulations. The TSC will meet biannually to assess study progress, review interim data if needed, and discuss any protocol deviations or necessary amendments. The TSC comprises an independent chairperson, clinical experts in obstetrics and gynecology, a statistician, an ethics advisor, and the principal investigator. Most members are independent, ensuring objective oversight of the trial.

The Data Management Team, integrated within the coordinating center, will oversee data entry, coding, and storage, ensuring the accuracy and security of the data.

No additional committees, such as an endpoint adjudication committee or data monitoring committee, are planned. The study team and the TSC will collectively handle oversight of key outcomes and adjudication, as needed.

### Composition of the data monitoring committee, its role and reporting structure {21a}

Given the low-risk nature of the intervention and the multi-center design of this study, a formal DMC will not be established. Instead, oversight of safety monitoring and interim analyses will be managed by the TSC. Oversight and decision-making responsibilities, particularly those related to safety and data integrity, are adequately covered by the TSC.

### Adverse event reporting and harms {22}

The study team will collect, assess, report, and manage any adverse events (AEs) or unintended effects related to the trial intervention. Adverse events will be classified as either solicited (actively monitored during the study) or spontaneously reported by participants.

All reported AEs will be documented in the participant’s CRF and assessed for severity, causality, and expectedness. Serious adverse events (SAEs), such as those resulting in hospitalization, disability, or death, will be reported to the TSC and the relevant ethics board within 24 h of occurrence.

The TSC will review all AEs and SAEs during their quarterly meetings, but they may convene earlier if serious safety concerns arise. If necessary, the TSC will recommend modifications to the study protocol or trial termination based on the severity or frequency of AEs.

### Frequency and plans for auditing trial conduct {23}

This trial will undergo regular internal monitoring and periodic external auditing to ensure compliance with the study protocol, regulatory requirements, and ethical guidelines. The Trial Coordinating Centre at IVFHK-CUHK will conduct quarterly internal monitoring at all participating sites (PWH-CUHK, CUHK Medical Centre, HEAL Fertility) to review informed consent procedures, protocol adherence, data integrity, and adverse event reporting. Findings will be reported to the TSC for review and corrective action if needed. Additionally, if required by regulatory bodies or ethics committees, an independent external audit may be conducted annually or upon request to assess compliance with Good Clinical Practice (GCP) and institutional guidelines. External audits will be independent of the investigators and the sponsor and will include a review of trial documentation, participant safety records, and data management processes across all sites. All monitoring and audit findings will be documented, reported, and addressed promptly to maintain trial integrity and regulatory compliance.

### Plans for communicating important protocol amendments to relevant parties (e.g., trial participants, ethical committees) {25}

Any significant protocol amendments, such as changes to eligibility criteria, outcomes, or analysis methods, will be communicated to all relevant parties, including investigators, ethics committees, trial participants, trial registries, and regulatory authorities. The sponsor and funders will be notified about the amendments first. The coordinating PI will then allocate the revised standard operating protocol to each participating center. Amendments will be documented, approved by the appropriate bodies, and participants will be re-consented if necessary. The protocol amendments will also be updated in the clinical trial registry. Additionally, any deviations from the protocol will be recorded using a breach report form.

### Dissemination plans {31a}

The results of the trial will be communicated to participants, healthcare professionals, and the public through peer-reviewed publications and presentations at scientific conferences. Trial outcomes will also be reported in relevant clinical trial registries. Participants who wish to receive the study results will be provided with a summary upon request. There are no publication restrictions or limitations on data sharing.

## Discussion

This trial protocol presents the design of an RCT comparing the novel direct warming method to the conventional multi-step warming process for FET. The trial seeks to determine whether this simplified method, which eliminates cryoprotectants and shortens the warming process, offers comparable clinical outcomes in terms of embryo survival, pregnancy rates, live birth rates, or any adverse events.

Given that maternal age is a well-established factor affecting fertility outcomes, participants are stratified into defined age groups [17]. This stratification ensures that age-related differences in embryo quality or implantation potential are accounted for in the analysis, enhancing the rigor and validity of the results.

The novel direct warming method offers significant advantages. Unlike other ultra-fast warming methods that use cryoprotectants such as sucrose or trehalose to prevent osmotic stress [7, 8], our method eliminates the need for cryoprotectants altogether. This reduces the complexity of the process, minimizes the risk of cryoprotectant-related toxicity, and significantly reduces the time required for warming, from approximately 20 min to just 3 min. This efficiency could optimize clinical workflows, particularly in high-volume fertility clinics, while reducing costs by eliminating the need for multiple thawing solutions.

Our simplified approach directly challenges the conventional understanding of embryo warming, which emphasizes multi-step processes to gradually remove cryoprotectants [12, 18]. By demonstrating that a single-step approach can achieve comparable embryo survival and pregnancy outcomes, this trial has the potential to shift the current paradigm. While some may argue that the absence of cryoprotectants increases the risk of osmotic stress, our preliminary data suggest that this risk can be effectively managed without compromising embryo viability or pregnancy rates [9, 19]. This novel method also offers practical advantages in terms of standardization and ease of use, which could make it an attractive option for fertility clinics aiming to streamline their protocols.

However, as a multi-center study, the findings may still be influenced by site-specific variations in clinical practices and patient demographics, which could impact generalizability. While the inclusion of multiple centers enhances the study’s external validity, further large-scale trials in diverse settings will be necessary to confirm broader applicability. Additionally, while miscarriage, pregnancy, and live birth rates are primary outcomes, long-term follow-up on birth outcomes, including potential congenital abnormalities, will be crucial to fully assess the safety of this novel method.

This trial protocol is positioned to provide valuable insights into whether the direct warming method can serve as a more efficient and equally effective alternative to conventional multi-step warming. If successful, it could simplify clinical protocols, reduce operational costs, and challenge the traditional understanding of embryo warming processes in ART.

## Trial status

This protocol is for Study Protocol Version 2, finalized on March 8, 2024. Participant recruitment is scheduled to begin on November 1, 2025, and is expected to be completed by October 31, 2028. The study is preparing for recruitment, and ongoing updates will be provided as the trial progresses.

## Data Availability

The final trial dataset will be accessible only to the principal investigator (PI) and designated research team members directly involved in the data analysis. These individuals will be responsible for ensuring the accuracy, integrity, and confidentiality of the data. No contractual agreements limit access to this dataset for the investigators, and all relevant personnel will follow standard data protection protocols to maintain participant confidentiality. Data sharing beyond the research team will require approval from the ethics committee, ensuring adherence to data privacy regulations. The availability of data for future use will also depend on institutional and regulatory guidelines, in compliance with data-sharing policies.

## Abbreviations

AEs: Adverse events
ARTs: Assisted reproductive technologies
CEA: Cost-effectiveness analysis
CI: Confidence intervals
CREC: Clinical Research Ethics Committee
CRFs: Case report forms
CRP: Clinical pregnancy rate
CUHK: The Chinese University of Hong Kong
CUHKMC: CUHK Medical Centre
DMC: Data Monitoring Committee
DMT: Data Management Team
EAC: Endpoint Adjudication Committee
FET: Frozen embryo transfer
GCP: Good Clinical Practice
GLM: Generalized linear models
LBR: Live birth rate
ITT: Intention-to-treat
IVF: In vitro fertilization
OPR: Ongoing pregnancy rate
OR: Odds ratios
PI: Principal investigator
PWH: Prince of Wales Hospital
RCT: Randomized controlled trial
RIF: Repeated implantation failure
RM: Recurrent miscarriage
SAEs: Serious adverse events
SET: Single embryo transfer
TSC: Trial Steering Committee
